# Oxygenated right ventricular assist device as part of veno-venopulmonary extracorporeal membrane oxygenation to support the right ventricle and pulmonary vasculature

**DOI:** 10.1186/s13019-023-02264-8

**Published:** 2023-04-11

**Authors:** Asad Ali Usman, Marisa Cevasco, Marc O. Maybauer, Audrey Elizabeth Spelde, Salim Olia, Christian Bermudez, Michael Ibrahim, Wilson Szeto, William J. Vernick, Jacob T. Gutsche

**Affiliations:** 1 Department of Anesthesiology and Critical Care, University of Pennsylvania, Hospital of the University of Pennsylvania, 3400 Spruce St, 6 Silverstein Pavilion, Philadelphia, PA USA; 2 Department of Surgery, Division of Cardiovascular Surgery, University of Pennsylvania, Hospital of the University of Pennsylvania, Philadelphia, PA USA; 3 Advanced Cardiac and Critical Care, Nazih Zuhdi Transplant Institute, 24/7 Shock Service, Intergris Baptist Medical Center, Oklahoma City, OK USA; 4 Department of Anaesthesiology and Intensive Care Medicine, Philipps University, Marburg, Germany; 5 Critical Care Research Group, The Prince Charles Hospital, The University of Queensland, Hospital cardiac Arrest, Brisbane, Australia

**Keywords:** Right ventricular assist device, Protek Duo, RA-PA cannula, Dual Lumen Cannula, Acute Right Ventricular failure, Mechanical circulatory support, Echocardiography

## Abstract

COVID–19 infection can lead to severe acute respiratory distress syndrome (ARDS), right ventricular (RV) failure and pulmonary hypertension. Venovenous extracorporeal membrane oxygenation (V-V ECMO) has been used for patients with refractory hypoxemia. More recently dual-lumen right atrium to pulmonary artery oxygenated right ventricular assist devices (Oxy-RVAD) have been utilized in the severe medical refractory COVID ARDS setting. Historically, animal data has demonstrated that high continuous non-pulsatile RVAD flows, leading to unregulated and unprotected circulation through the pulmonary vessels is associated with an increased risk of pulmonary hemorrhage and increased amount of extravascular lung water. These risks are heightened in the setting of ARDS with fragile capillaries, left ventricular (LV) diastolic failure, COVID cardiomyopathy, and anticoagulation. Concurrently, due to infection, tachycardia, and refractory hypoxemia, high V-V ECMO flows to match high cardiac output are often necessary to maintain systemic oxygenation. Increase in cardiac output without a concurrent increase in VV ECMO flow will result in a higher fraction of deoxygenated blood returning to the right heart and therefore resulting in hypoxemia. Several groups have suggested using a RVAD only strategy in COVID ARDS; however, this exposes the patients to the risk of pulmonary hemorrhage. We present one of the first known cases using an RV mechanical support, partial flow pulmonary circulation, oxygenated Veno-venopulmonary (V-VP) strategy resulting in RV recovery, total renal recovery, awake rehabilitation, and recovery.

## Introduction

Severe acute respiratory distress syndrome (ARDS) can be caused by a number of inciting factors including COVID-19, bacterial pneumonia, pancreatitis, and trauma [1, 2]. Conventional management for ARDS includes prone positioning, low stretch ventilation, and deep sedation. Venovenous extracorporeal membrane oxygenation (V-V ECMO) can be used in severe cases of ARDS. Thus far 14,861 COVID patients worldwide have been managed with ECMO and in-hospital mortality is 47% while non-COVID ECMO mortality is approximately 37.1% [3, 4]. Typically, a standard internal jugular-femoral vein (IJ-Fem) cannulation or dual-lumen single cannula (DLSC) V-V ECMO strategy has been used in ARDS ECMO [5]. V-V ECMO, depending on the cannulation configuration, can have varying degrees of recirculation depending on the flow rate, proximity of the inflow to outflow cannula(s), cannula location, and cannula size. It is important to note that impaired RV physiology occurs in up to 20% of patients with ARDS and is a major determinant of recirculation and mortality [2, 6].

A high incidence of acute and subacute RV failure has been observed in COVID ARDS, secondary to dramatic increases in pulmonary vascular resistance due to hypoxia, hypercarbia, lung injury. Furthermore, RV strain occurs during attempted sedation weaning when profound coughing, subjective feelings of suffocation, shortness of breath, and fluctuations in pressures can occur with rapid breathing with high inspiratory pressures and desynchrony. This can associated increased intrathoracic pressures, mechanical ventilation weaning, and clinical and subclinical pulmonary emboli associated with COVID infection [7, 8]. RV failure can primarily manifest as acute kidney or liver injury, RV dilation and RV systolic failure, worsening hemodynamics, and increasing vasoactive and inotropic medication requirements [9]. Patients with severe COVID ARDS can also have inducible pulmonary hypertension during wake up trials, in those patients with difficulty during sedation weaning, concurrent to ventilator induced lung injury, which can result in VV ECMO recirculation. Abnormal interaction between the RV and pulmonary vasculature in ARDS is associated with adverse clinical outcomes [10, 11]. RV mechanical circulatory support bypasses blood from the RA to the PA. Oxygenated right ventricular devices (Oxy-RVAD) using a dual-lumen right atrium to pulmonary artery has been used in COVID ARDS with and without right ventricular failure [1, 12−14].

Oxygenated RVAD can be used to bypass the failing right ventricle and directly introduce oxygenated blood directly into the pulmonary artery. As found in the animal model, One of the limitations of high RVAD flow is pulmonary edema and hemorrhage, however this was not borne out in high rates of usage of Oxy-RVAD in COVID ARDS [14–17]. Full flow continuous RVAD devices may lead to these complications in several scenarios: when LV systolic function drops below RVAD flows, in the setting of LV diastolic failure, mitral stenosis, or pulmonary venous occlusive disease [17, 18]. Furthermore, a high rate of stasis and RV clot may occur if the RV is not washed with blood during full flow RVAD support. Given this understanding, we followed the strategy of deploying RV mechanical circulatory support while simultaneously protecting the pulmonary circulation from high continuous flow overcirculation and providing flow through the RV using a partial RA flow Veno-venopulmonary artery (V-VP) ECMO strategy. Previously, this strategy has been used in short temporary duration, however; we report one of the first novel case of a near entire ECMO duration using a protected RV protected, pulmonary circulation partial flow, oxygenated Veno-venopulmonary (V-VP) RVAD strategy [19, 20].

## Case

**Fig. 1:**
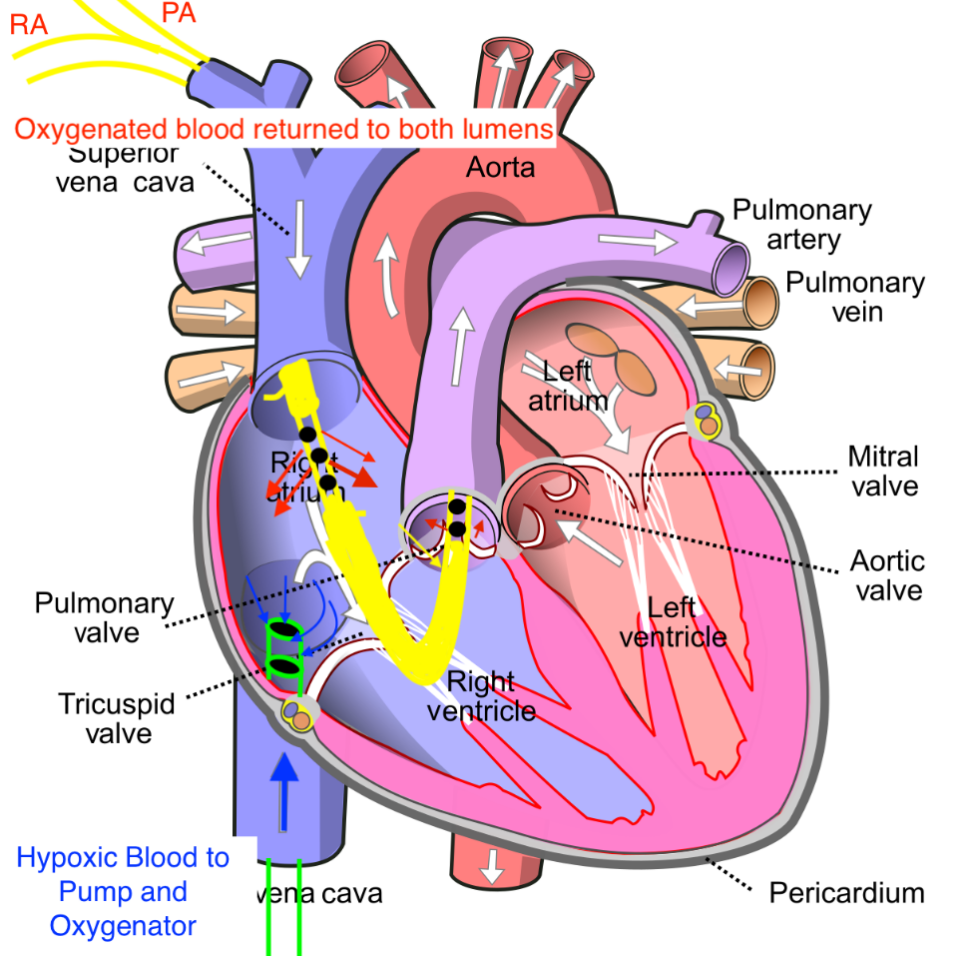
Diagram demonstrating oxygenation of both limbs of the dual-lumen Protek Duo cannula. Oxygenating the proximal limb creates a configuration similar to conventional venovenous ECMO while oxygenating the distal limb creates an oxygenated right ventricular assist device. This configuration is V-VP ECMO with split flow to the pulmonary artery and the right atrium

**Fig. 2:**
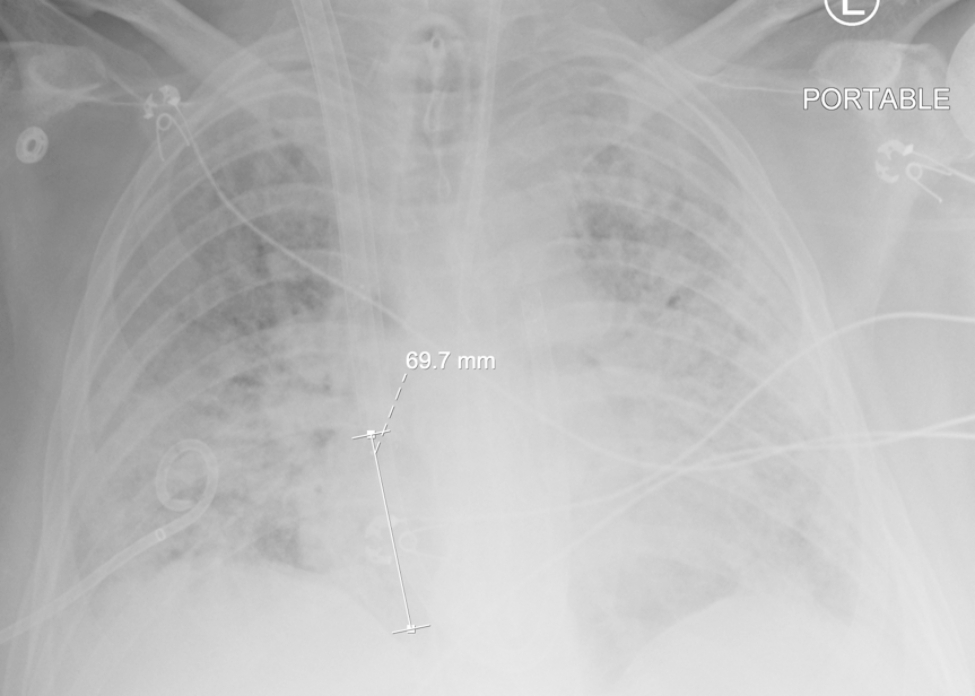
Dual-lumen single cannula as dual oxygenated outflow to the RA and RV. The distance between the RA limb and the femoral inflow cannula is 7 cm to minimize recirculation

**Fig. 3:**
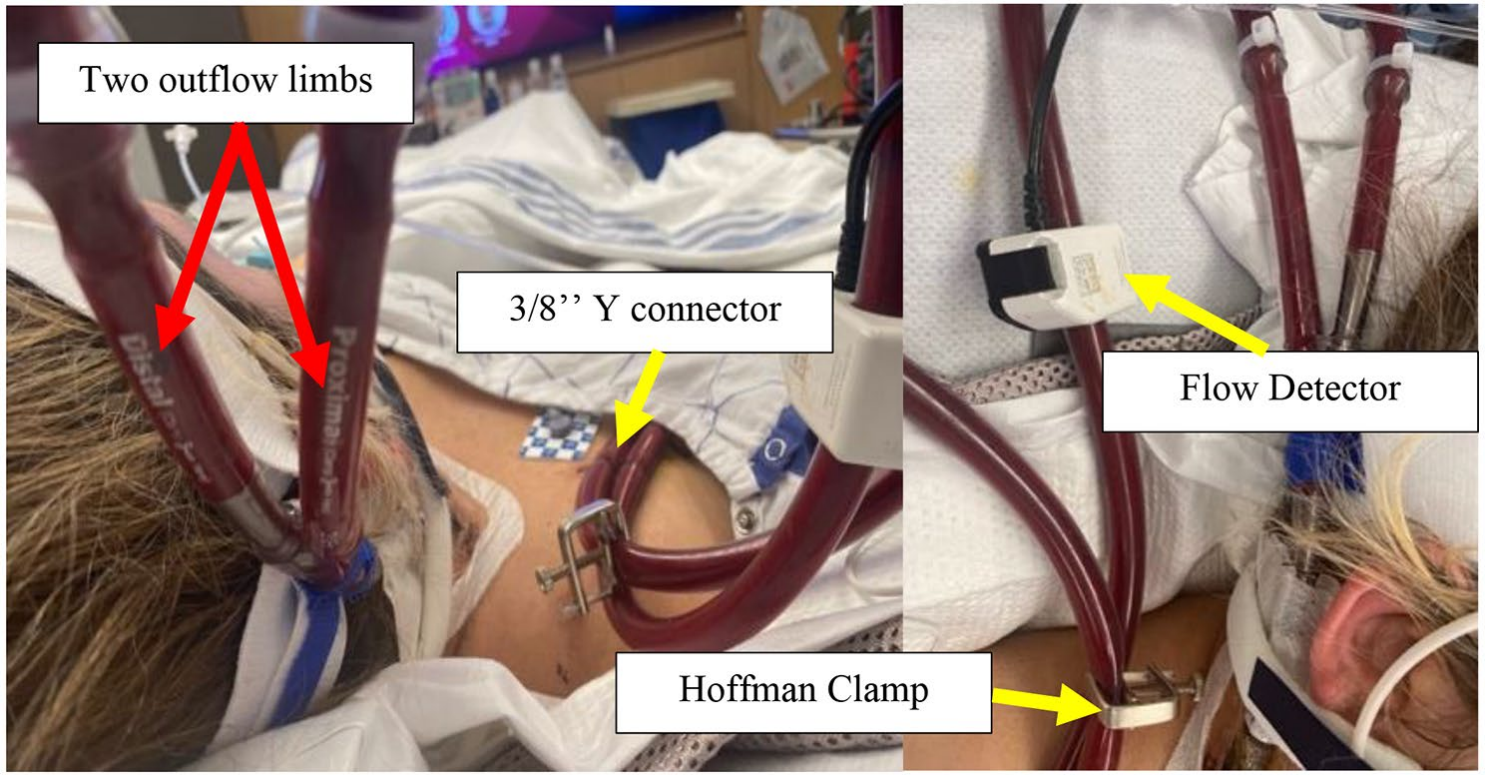
Dual oxygenated V-VP ECMO with flow sensor and clamp applied. The flow sensor is positioned on the distal limb to monitor the PA blood flow closely. The clamp is applied on the proximal low pressure RA limb to calibrate the flow appropriately to avoid pulmonary artery overcirculation. There is a 3/8’’ Y tubing connector splitting flows

The patient was a previously healthy 30-year-old male, BMI 30, BSA 2.1, who presented to an outside institution with COVID pneumonia. He was unvaccinated and received treatment with intravenous dexamethasone and baricitinib. His disease progressed into severe ARDS refractory to conventional treatment including initiation of mechanical ventilation, prone positioning, sedation and paralysis. After a total of 16 days in the hospital and 5 days of mechanical ventilation our team was consulted for mobile ECMO evaluation. At that time a CT chest was consistent with severe COVID ARDS and negative for pulmonary embolism. He was cannulated at the outside hospital with a 25 French Medtronic (Minneapolis, MN) right femoral inflow cannula and a 19 French Medtronic right internal jugular outflow cannula with transesophageal echocardiography (TEE) guidance in the intensive care unit. At the time of cannulation TEE demonstrated moderate RV dilation, moderate RV systolic dysfunction with a TAPSE measured at 0.9 and FAC measured at 13%, and moderate tricuspid regurgitation. There was normal LV function estimated by visual assessment of EF of 60–65% and a normal mitral valve without mitral stenosis or mitral regurgitation. The PA was noted to be moderately dilated as well. The estimated PA systolic pressure based on tricuspid regurgitation jet was 67 mmHg. The patient was started and maintained on inhaled epoprostenol and intravenous epinephrine. The patient remained on these medications throughout the ECMO support course. Twenty-four hours after V-V ECMO initiation, he was noted to have acute kidney injury and liver injury with serum creatinine rising from 0.58 mg/dL on admission at our hospital to a peak of 3.5 mg/dL, and AST/ALT levels of 12,692/4,463 U/L, respectively. He became anuric and was initiated on continuous renal replacement therapy (CRRT). At this point there was clinical concern for acute RV failure. This was due to severely reduced RV systolic function on echocardiography with liver and renal failure and a central venous pressure of 22 mmHg, pulmonary capillary wedge pressure of 8 mmHg, and a PVR of 3.4 woods units. In this particular case, transthoracic echocardiography and right heart catheterization were used to confirm acute RV failure. The potential causes of acute RV failure included pulmonary embolism, progressive pulmonary edema and ARDS RV failure, COVID cardiomyopathy, or septic shock. Due to the aforementioned reasons, 24 hours after VV ECMO initiation, he was taken to the operating room for cannula reconfiguration to Oxy-RVAD. In the operating room, he was placed in the supine position and both groins and right neck were prepared. He was transitioned temporarily to femoral-femoral V-V ECMO using the existing femoral cannula and a new 22 French Biomedicus (Medtronic, MN) outflow cannula positioned high in the right atrium. The 19 French right IJ cannula was removed and a new 31 French Protek duo (Livanova, UK) cannula was placed. In the operating room the patient had a continuous cardiac output measurement of 6.5 LPM on epinephrine infusion of 8 mcg/min, vasopressin 0.04 units/min, as well as inhaled epoprostenol at 50 ng/min. The VV ECMO flow rate was 5.2 LPM and the patient SaO2 was 91%. At the time of Oxy-RVAD conversion we were concerned about needing to flow an Oxy-RVAD at 6.2 LPM to avoid circuit shunt and continued hypoxemia; all while being concerned about creating excessive pulmonary edema and possibly pulmonary hemorrhage. Therefore, to balance the need for high flows as well as RV mechanical support, we opted to place the patient in the novel RV protected, pulmonary circulation protected, split flow strategy. The ECMO outflow tubing was bifurcated with a 3/8’’ Y connecter, subsequently both proximal RA limb and distal PA limb of the Protek Duo cannula were connected to oxygenated outflow tubing. Therefore, the final configuration was femoral venous inflow to right atrium & pulmonary artery, split outflows, veno-venopulmonary artery configuration. (Fig. 1) The ECMO cannulas were connected to a Spectrum CP 22 Quantum (Chelttenham, England) centrifugal pump and a Maquet Getinge Quadrox-I (Rastatt, Germany) adult oxygenator. The distance between the prior 25 French femoral inflow cannula and the proximal holes on the Protek duo measured 7 centimeters. (Fig. 2) A flow probe was placed on the PA limb and a Hoffmann clamp was placed on the RA limb to adjust the flow rate of the two outflow limbs. (Fig. 3) The flow goals were defined to keep the PA limb flow no greater than 2.5 LPM while providing the rest of the total flow through the RA limb. This was enough flow to reverse the renal and liver injury. The target PaO_2_ was greater than 60 mmHg and pH > 7.2 with a PaCO_2_ of less than 60 mmHg. Over the course of V-VP ECMO he remained on 5.0–6.0 LPM total flow and the PA limb was limited to 2.5 LPM. The patient was anticoagulated with bivalirudin with a PTT goal of 55–65 s. Daily management of the ECMO circuit was performed by the ECMO team (AAU, AES, WJV, JTG) on daily rounds which included oxygenator and tubing visual inspection, assessment of daily hemolysis labs including D-Dimer, Fibrinogen, Lactate Dehydrogenase as well as titration of PTT goals. The patient remained on CRRT for three additional weeks after V-VP conversion in which he started to gradually recover renal function with increased urine output. On day 22 Post V-VP conversion was weaned from CRRT while remaining on ECMO. While sedated the patient failed to recover lung function with compliance ranging from 7 to 11 ml/cmH_2_0. Tidal volumes remained below 100 cc on low stretch ventilation which was defined as PEEP < 10 cmH_2_0, respiratory rate less than 16, and peak airway pressures targeted to remain no higher than 30 cmH_2_0. The RV split flow allowed for a protected sedation wean in spite of large fluctuations in intrathoracic and pleural pressure during wake up. He eventually was weaned from all continuous infusion sedation and participated in 4 weeks of ambulatory awake rehabilitation on V-VP ECMO. Serial weekly transthoracic echocardiography was performed which demonstrated significant remodeling of RV size and improvement of RV function to normal. The TR Vmax estimate decreased to 29 mmHg suggesting resolution of pulmonary hypertension and central venous pressure was calculated as 8 mmHg. Pre- and post-oxygenator blood gas, transmembrane pressure monitoring was done daily. The PaO2 with goal was set at < 55 mmHg and pH at 7.2. There was minimal recirculation fraction. He was treated with cefepime until resolution of the superimposed bacterial pneumonia was resolved and the covid cycle threshold was below detectable. The COVID ARDS had resolved. After a total of 51 days of V-VP ECMO support he was bridged back to left internal jugular single lumen reinfusion catheter with the existing femoral venous inflow cannula to V-V ECMO due to clot formation in the V-VP outflow venous limb. During ambulatory oxy-RVAD support the patient’s compliance gradually improved to 52 ml/cmH_2_0. At this point he was transitioned to pressure support ventilation with intermittent tracheostomy collar trials. At ECMO day 64 he was transitioned off ECMO with decannulation and placed on a HemoLung for 16 days (Alung, Pittsburg) for continued CO_2_ removal. He required a total of 6 circuit exchanges for the duration of ECMO support and discharged after 86 days in the hospital.

## Discussion

The three most common configurations of V-V ECMO used in ARDS are Femoral-IJ, bicaval, and bifemoral. All three configurations return oxygenated blood to the right atrium. Systemic oxygenation while on ECMO is dependent on several factors including tricuspid valve competence, RV systolic function, RV afterload, pulmonary vascular resistance, mitral valve competence, LV diastolic and systolic function, and total cardiac output. RV failure and pulmonary hypertension can occur in up to 20% of patients with ARDS, and is likely higher in patients requiring V-V ECMO support [2]. RV failure occurs due to two distinct processes: direct RV systolic failure and increases in RV afterload/impedance. Impedance is directly related to heart rate, viscoelastic properties of the vessel, and wave reflections. The changes in impedance resulting from large-artery stiffening or remodeling alone can markedly alter the load on the RV. In ARDS, increases in RV afterload occurs due to hypoxia, pulmonary edema, pulmonary embolism, and extreme ventilator settings with high PEEP and airway pressures. RV afterload and pulmonary vascular resistance is dynamic and can change based on various physiological states including attempted sedation weaning, ventilator weaning trials, and changing oxygenation and hypoxic pulmonary vasoconstriction effects.

Acute RV failure is common in severe COVID ARDS [7, 8]. To address RV failure, several groups have converted patients who are on VV ECMO to oxygenated RVAD support or venoarterial ECMO.(Table 1) [1, 12−14]. Some of the limitations of VA ECMO are the need for arterial access as well as the higher rates of complications associated with arterial access including bleeding, leg ischemia, and stroke [21]. Furthermore, in COVID ARDS patients typically require longer-term support and in the setting of VA ECMO this would be a prohibitive platform and therefore Oxy-RVAD may represent a better solution for prolonged support. In this case, our patient had a cardiac output of 6.5 LPM on continuous cardiac output monitoring and a VV ECMO flow rate of 5.2 LPM with progressive RV failure and refractory hypoxia. There is theoretical concern using a pure RVAD strategy may result in excessive pulmonary edema and pulmonary hemorrhage if the RVAD flows are greater than what the LV can tolerate. Our patient had a VV ECMO flow rate of 5.2 LPM. When converting the patient to an oxy-RVAD we were concerned that the required high flow may lead to developing pulmonary edema and pulmonary hemorrhage. While on VV ECMO matching the ECMO flow at least 60% of the cardiac output is necessary to achieve adequate systemic oxygenation [22]. This study in animals, is not completely similar to full oxy-RVAD support clinically because presumably there remains some fraction of pulsatile flow through circuit shunt. Second, there is limited data to suggest that altering the pressure pulsatility, and lack of pulsatile flow, as well as increasing the pulmonary artery pressure with continuous flow RVADs may contribute to the risk of lung edema hemorrhage. Teguchi et al. demonstrated that non-pulsatile pulmonary perfusion during RVAD support can lead to increased lung water accumulation compared to pre-bypass due to increased endothelial permeability [23]. It is important to note that in this report the PaO2 did not differ between the pulsatile and non-pulsatile groups. Clinically, Welp et al. studied 25 patients who underwent LVAD with temporary RVAD placement. They found 5/25 patients developed pulmonary hemorrhage after 7 days of RVAD support, particularly those patients requiring flow greater than 4 LPM [17]. Furthermore, bronchoscopy in patients with full flow RVAD support demonstrated vulnerable bronchial mucosa when in contact with the bronchoscope likely due to pulmonary capillary congestion [17]. Several groups have explored best methods in calibrating RVAD flows by applying banding or ligation of the pulmonary artery to titrate RVAD afterload and subsequently measure RA and LA flows [18]. They have found that the LA pressures acutely changed with excessive RVAD flows, however this does not apply to LVAD flows transmitting to the RA likely due to a higher capacitance of the systemic venous system [18]. Oda et al. also studied calibration of RVAD flow to LVAD flow and demonstrated that a R/L ratio of < 0.9 resulted in increased survival. This data all suggests that lower RVAD flows should be used to prevent pulmonary hemorrhage, edema, and overcirculation [24]. Although the clinical data is limited in how to appropriately titrate RVAD flow, we elected to place the patient on a V-VP ECMO platform which may provide a balanced approach addressing RV failure with RV protection and avoiding pulmonary overcirculation (clear CXR and lung ultrasound without b-lines) protection with partial PA flow. Further clinical research is necessary to identify appropriate patient selection for RVADs and titration of RVAD flows. Extrapolating the existing published animal data and clinical BiVAD data may not directly apply to the COVID ARDS setting, and our patient may have clinically improved in spite of the V-VP OxyRVAD configuration.

**Table 1:** Three different ECMO support options for patients with ARDS and RV failure with listed pros and cons

Configuration	V-P ECMO	V-VP ECMO	VA ECMO
**Inflow**	Right atrium (Protek Duo or two separate cannulas) Right Atrium + Right Ventricle (Spectrum Cannula)	Inferior Vena Cava	Right Atrium
**Outflow**	Pulmonary Artery	Right Atrium and Pulmonary Artery Split Flows	Femoral Artery
**Cannulas**	Protek Duo (Livanova, UK) 31 French or 29 French Spectrum (Cheltenham, UK) 31 French or 27 French Biomedicus two separate cannulas (Medtronic, MN) 17–21 French outflow and 23–25 French inflow	Protek Duo with 3’8’’ Connector as both limbs are oxygenated. Only possible with Protek Duo Need separate Femoral venous inflow 23–25 french cannula	23–25 French Internal jugular or femoral venous inflow 15–17 French femoral arterial or axillary artery (sport Mode) outflow
**Advantages**	- Complete RV support - Can use dual-lumen single cannula and have only neck cannulation possible with Spectrum or Protek Duo Cannula which increases mobility	Partial RV support Maintain PA pulsatility Avoid excessive flow in the PA	Biventricular support
**Disadvantages**	Potential increased risk of pulmonary edema or hemorrhage if there is a drop in LV function, LV diastolic failure, Mitral stenosis, pulmonary embolism	- Increased risk of thrombosis in the lower flow limb Possible - Recirculation between the V-V limb - Requires femoral cannula and can limit mobility	- Leg ischemia and limited by duration of support - Increased risk of stroke and bleeding. - Potentially requires surgical cut down for arterial cannula removal.

None the less, given this background on the deleterious effects of high RVAD flows we sought to create a solution to RV failure in COVID ARDS in those patients still requiring high flows for hypoxemia. We balanced the requirement for RV mechanical support with the opposing high flows necessary for oxygenation. This strategy involved placing a dual-lumen Protek Duo (Livanova, USA) RA – PA catheter in the right internal jugular vein. We then used a separate femoral venous cannula as our ECMO inflow. The ECMO outflow tubing was bifurcated and connected to both limbs of the Protek Duo. We calibrated the flows based on end-organ dysfunction and resolution of liver failure and renal failure. There is are reports of V-VP ECMO configuration that were used for a partial duration of total ECMO support [18–20]. In these cases, described by Maybauer and colleagues, all patients were either on V-V ECMO or V-P ECMO before the configuration was changed to V-VP ECMO without any complications.

Due to the lower pressure RA, in comparison to the PA, an external clamp was applied to the RA-proximal limb in order to calibrate flow to the PA limb. A second flow probe was applied to the PA-distal limb and RVAD supported flows were kept at a strict 2.5 LPM. This allowed a degree of native PA pulsatility while simultaneously offloading the RV without pulmonary overcirculation. However, the small diameter and length of the distal limb may not allow for much more flow than 3 LPM. Usually 60–70% of blood flows through the proximal limb. Clinicians should weigh out the risk-benefit-ratio of clamping the tubing for a longer time-period, since hemolysis and clotting may occur with the inline Hoffman clamp [25]. The proximal – RA outflow orifice was utilized as IJ-Fem V-V ECMO configuration. The separation between the proximal limb and the femoral cannulas was 7 centimeters as measured on supine anteroposterior chest radiography which limits recirculation. Total ECMO flows was maintained at 5.0 LPM to 6 LPM as necessitated by the degree of hypoxemia and oxygen saturations. Using the ELSO nomenclature this configuration should be called V-VP ECMO, as a protecting strategy for RV failure and split PA flow to avoid pulmonary overcirculation [5]. Using this strategy, we were able to fully recover renal function successfully discontinuing continuous renal replacement therapy well as recover liver function. With this strategy, the patient was able to wean from sedation and ambulate without progressive RV failure while tolerating fluctuations in intrathoracic pressure and dynamic pulmonary hypertension due to severe intermittent coughing.

Drawbacks to this cannulation configurations include increased risk of lumen thrombosis and flow limitations. To mitigate this risk, we used the CP22 Quantum Spectrum centrifugal pump; however, a Centrimag (Levitronix) pump may have further reduced the risk of thrombosis. Likewise, we used a Quadrox-I oxygenator however; other oxygenators such as the Medtronic Nautilus oxygenator which is also highly rated, may have reduced the risk of thrombosis. When assessing the quality and function of various oxygenators, consideration should be paid to the pressure-flow (HQ) curves of the oxygenator, the pressure drops across the membrane lung, the efficiency of oxygenation at various flow rates, the CO2 transfer rate per blood flow rate, and the heat exchange factors. Furthermore, there should be close short term follow up for residual valvular complications post decannulation. Cannulas that traverse the tricuspid and pulmonic valve can lead to valvular incompetency and short-term thrombus formation in the right atrium and right ventricle. In a case series of 16 RA to PA cannulas, Usman et al. describe 4 patients with echodensities found in the RA and TV at 14.7 days post decannulation on surveillance echocardiography [26].

## Conclusion

We present a novel case of V-VP ECMO. In this configuration oxygenated blood is returned through both limbs of the Protek Duo cannula. While supported on this strategy our patient had resolution of ARDS, recovery from RV failure, renal and liver recovery with weaning of CVVH, sedation free ECMO support and ambulatory rehabilitation, with an outcome as bridge to successful recovery. With this strategy we may have been able to mitigated the risk of pulmonary over circulation by limiting PA flows while protecting the RV with a RVAD mechanical circulatory support device. Although our VVP ECMO strategy is highly speculative and a pure oxy-RVAD configuration may not have led to increased pulmonary hemorrhage or edema, this novel configuration adds to the armamentarium available to the clinician managing RV failure in ARDS.

## Data Availability

Not Applicable.
